# Case report: Conquer a complex variant: Coronary-pulmonary artery fistulas, atrial septal defect and bicuspid pulmonary valve, under beating heart surgery

**DOI:** 10.3389/fcvm.2022.915874

**Published:** 2022-10-11

**Authors:** Ting Zhou, Chaobing Liu, Songlin Zhang

**Affiliations:** ^1^Department of Cardiothoracic Surgery, The First College of Clinical Medical Science, China Three Gorges University, Yichang, China; ^2^Department of Cardiothoracic Surgery, Yichang Central People’s Hospital, Yichang, China

**Keywords:** CPAF, on-pump beating heart surgery, ASD, bicuspid pulmonary valve, moderate tricuspid regurgitation

## Abstract

Coronary artery to pulmonary artery fistula (CPAF) is a congenital or acquired abnormal channel between arteries, with a left-to-right cardiac shunting, which may lead to myocardial ischemia, arrhythmia, thrombotic complications, and heart failure. CPAF is usually detected by coronary angiography but few reports have used beating-heart surgery as a detection method. The patient in this case report is a 39-year-old male diagnosed with atrial septal defect (ASD), bicuspid pulmonary valve, and moderate tricuspid regurgitation (TR). He is asymptomatic. In preoperative evaluation, significant CPAF was suspected using echocardiography. The patient refused coronary angiography due to allergic history. Therefore, the cardiac team designed and performed on-pump beating-heart surgery (OPBHS) to detect and repair these disorders, and suggested OPBHS as a myocardial protection strategy for the patient at low surgical risk. A rare and complex cardiovascular case with CPAFs from two branches of the left anterior descending coronary (LAD) artery to the main pulmonary artery (MPA) with ASD, bicuspid pulmonary valve, and moderate TR has not yet been reported in the literature, and its embryological hypothesis has been further analyzed in this report.

## Introduction

Coronary artery to pulmonary artery fistula (CPAF) is a congenital or acquired abnormal channel between the coronary artery and the pulmonary artery with a left-to-right cardiac shunting, which may lead to myocardial ischemia, arrhythmia, thrombotic complications, and heart failure (1, 2). From the reported literature, coronary to PA fistula comprises 16% of all coronary cameral fistulae. Coronary cameral fistulae have been reported in 0.1–0.5% of routine coronary angiograms and the vast majority of them are small and asymtomatic (2, 3).

We report a rare congenital cardiovascular variant with two CPAFs from two branches of the left anterior descending coronary (LAD) to the main pulmonary artery (MPA), coexisting with a bicuspid pulmonary valve and atrial septal defect (ASD), which has not yet been reported in the literature.

The patient was a 39-year-old male diagnosed with ASD, bicuspid pulmonary valve, and moderate TR, and he was asymptomatic. In the preoperative evaluation using echocardiography, a hemodynamically significant coronary-PA fistula was highly suspected. The patient refused to undergo a coronary angiography due to their allergic history to penicillin. This unique situation provided diagnostic and therapeutic challenges to the surgical team. As such, the cardiac team designed and performed OPBHS to detect and repair the CPAFs, ASD, bicuspid pulmonary valve, and moderate TR.

We present the following case in accordance with the CARE Reporting checklist (available at http://dx.doi.org/10.21037/acr-20-100).

## Case report

**FIGURE 1:**
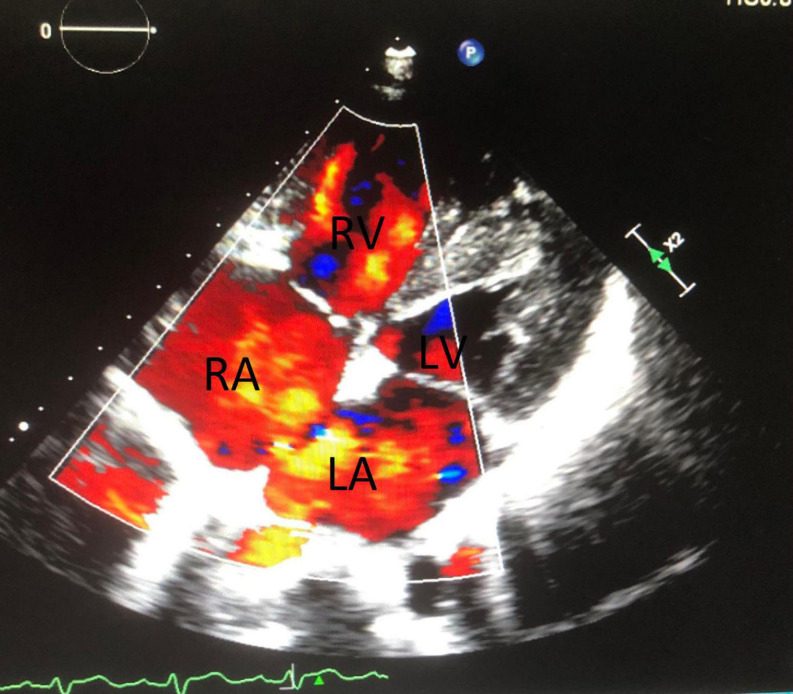
Shows the atrial septal defect.

A 39-year-old male, went to see a doctor with a cold, and a systolic ejection murmur (Levine 2 of 6) over his left upper sternal border was detected. Therefore, he was referred to our tertiary medical center for further evaluation. He was asymptomatic. His past medical history was paroxysmal hypertension, and he was an active smoker, without a family history of heart disease. Clinical examination suggested ASD with a moderate left-to-right shunt and no pulmonary artery hypertension (PAH). The laboratory tests were within the normal range and included: cardiac enzymes, N-terminal pro-B-type natriuretic peptide, inflammatory markers, lipid panel, and coagulation function. The 12-lead electrocardiogram (ECG) showed a sinus rhythm and a complete right bundle branch block (CRBBB), without signs of compromised myocardial perfusion or left ventricular hypertrophy. A chest CT scan reported a widening of the left and right pulmonary arteries and minor fibrosis of both lungs. Transthoracic echocardiography (TTE) (Figure 1) revealed a secundum ASD with a diameter of 25 mm, a bicuspid pulmonary valve, a widening of the pulmonary artery, and the right atrium and ventricle were dilated.

**FIGURE 2:**
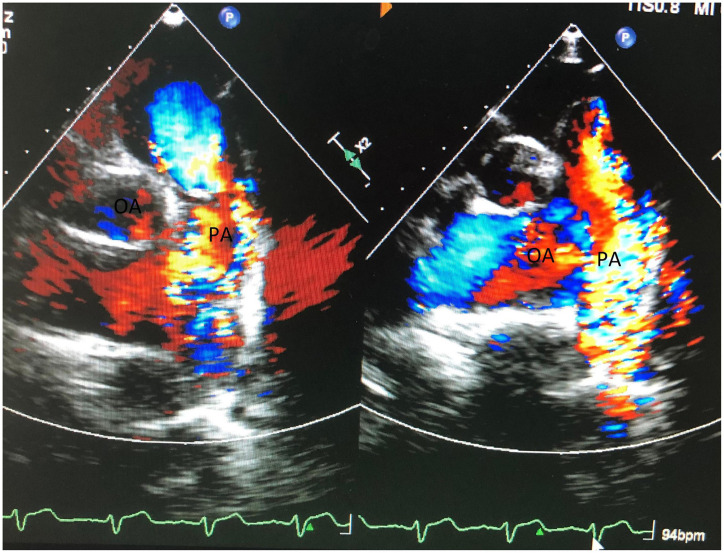
Shows obvious high velocity continuous and dominant diastolic aliasing color flow Doppler signals in MPA.

Additionally, transesophageal echocardiography (TEE) showed moderate tricuspid regurgitation (TR). Furthermore, TEE showed obvious high velocity continuous and dominant diastolic aliasing in the color flow Doppler signals in the MPA (Figure 2). The logical conclusions were that there might be either a small AP window, anomalous coronary from PA, coronary PA fistula, or atypical PDA. Combining the image of a large proximal left anterior descending artery (LAD) at origin, the surgical team suspected the diagnosis was LAD to PA fistula. Nevertheless, a transthoracic/transesophageal echo has the rare ability to visualize clear imaging of the fistulous connection. Furthermore, due to allergic history to penicillin, the patient refused to undergo coronary angiography.

**FIGURE 3:**
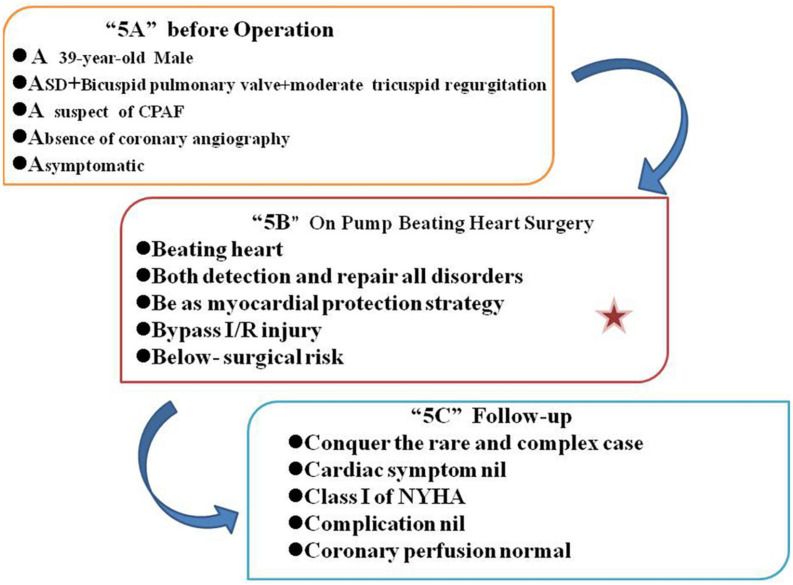
Total procedures.

After careful preoperative evaluation, there were three reasons for the choice of on-pump beating-heart surgery (OPBHS). First and foremost, to investigate the continuous aliasing of the color Doppler signals in the MPA, we can directly detect any coronary-PA fistula under beating-heart surgery. Furthermore, if the coronary –PA fistula existed, it could be treated effectively along with the ASD, bicuspid pulmonary valve, and moderate TR. Finally, considering the myocardial protection, OPBHS might reduce the risk of ischemia and reperfusion (I/R) injury. The total procedure was displayed in the Figure 3.

## Operative procedure

Through a small incision, using a median sternotomy, the OPBHS was performed. After opening the pericardium, the proximal segment of the LAD was about 10 mm in diameter and tortuous. Two branches of the LAD terminated at the trunk of the pulmonary artery (PA). The diameters of the two branches were 8 and 7 mm, respectively. The width of the MPA is about 30 mm.

First, the secundum ASD was repaired, with a 25*20 mm polyester patch. The MPA was opened through a transverse incision. Under the beating heart, two fistulas were found, just above the pulmonary valve in the MPA, with two left-to-right cardiac shunts. The size of the orifices of the two fistulas was 8 and 7 mm, respectively. They originated from two branches of the LAD that drained into the MPA. The lengths of their communications were 30 and 35 mm, respectively. The diameter of fistulous tracks was 8 and 7 mm, respectively. Therefore, coronary-pulmonary artery fistulas were large and significant and warranted surgery.

The heart team evaluated the condition of the patient and informed their authorized person of the unexpected finding of coronary to PA fistulae requiring surgical correction. After obtaining consent, the procedure was carried out. Two fistulous tracts arising from the LAD were traced, draining to the MPA, and clamped. The ECG monitor showed no ST changes so the left ventricular contractility remained unchanged. A surgical ligature of the two fistulous tracts was performed. The orifices of the fistulous tracts were closed with a 4-0 polypropylene suture from within the MPA.

The repairs of the bicuspid pulmonary valve and tricuspid valve were performed uneventfully. A 20 mm-probe could easily cross the right ventricular outflow. No residual defects or regurgitation was detected on the TEE after intracardiac manipulation.

The post-operative period was uneventful. Post-operative investigations showed normal cardiac enzymes and normal ECG. An echocardiographic evaluation revealed normal cardiac LV function and no significant residua. The patient was reviewed after 2 months. He did not have any cardiac symptoms, was carrying out normal activities, and was in the New York Heart Association (NYHA) class I. He was satisfied with the surgery and its outcomes.

All procedures performed in studies involving human participants were in accordance with the ethical standards of the institutional and/or national research committee(s) and with the Helsinki Declaration (as revised in 2013). Written informed consent was obtained from the patient.

## Discussion

It is reasonable to assume the patient had a congenital coronary to PA fistula from two branches of the LAD to the MPA, associated with an ostium secundum ASD and bicuspid pulmonary valve. Based on the Hackensellner involution-persistence hypothesis (4), we propose that the two fistulas might be two persistent anlages from truncus arteriosus. Other congenital heart defects have been associated with coronary fistula, varying from 0.5 to 5%. A large flow in the fistulas may cause myocardial ischemia or infarction. Therefore, the surgery was deemed to be required (1, 2, 5).

We propose the potential origin and reason of the case. Although there is no family history, it might be possible that the patient carries some genetic mutations leading to this rare case. In recent research from the UK NHS Genomic Medicine Centre, a heterozygous GDF2 variant lead to vascular malformation such as hereditary hemorrhagic telangiectasia (HHT), and it might be one of the genetic mutations for coronary-pulmonary fistula (6). Mutations in the *RBM10* gene are a cause of ASD, and might be responsible for PA fistula, according to a molecular study by Han and his colleague (7).

To deeply understand this complex case, we analyzed the patient’s hemodynamic results, the moderate left-to-right ASD shunt overload, and abraded the tricuspid valve. Despite the additional volume from two Coronary-PA fistulas and ASD, under the obstruction of the bicuspid pulmonary valve, fortunately, the pulmonary artery gradually widened without PAH.

In our case, CPAFs were successfully detected and treated under OPBHS. The clinical advantages of the OPBHS included:(1) a near-physiologic state, maintaining continuous coronary blood flow avoiding ischemia and reperfusion injury; (2) CPAFs were identified and carefully evaluated under direct vision; and (3) ligature of two fistulous tracts was performed under direct vision, preventing downstream coronary steal and left-to-right shunt. The patient was easily weaned off CPB. No ST changes or significant arrhythmia were detected by ECG monitoring.

OPBHS was initiated as a myocardial protection strategy to prevent ischemia/reperfusion injury. It was first applied by Wei and his colleagues in 1993 for a patient undergoing mitral valve replacement (8).

Usually, OPBHS is reserved for high surgical risk with LV dysfunction/chronic kidney disease (9–13). With the outcomes of our case, this technique may be possible for patients with low surgical risk.

### Limitations

The surgical team summarized the experience of this case, analyzing the total diagnosis and treatment process. The preoperative assessment could be more comprehensive. A 39-year-old male, without coronary angiogram due to patient refusal, even though asymptomatic, with cardiac enzymes in the normal range, without signs of ischemia on ECG, and with variable coronary risk factors (hypertension, active smoker), could have a guarded coronary treadmill evaluation and myocardial perfusion scan.

**FIGURE 4:**
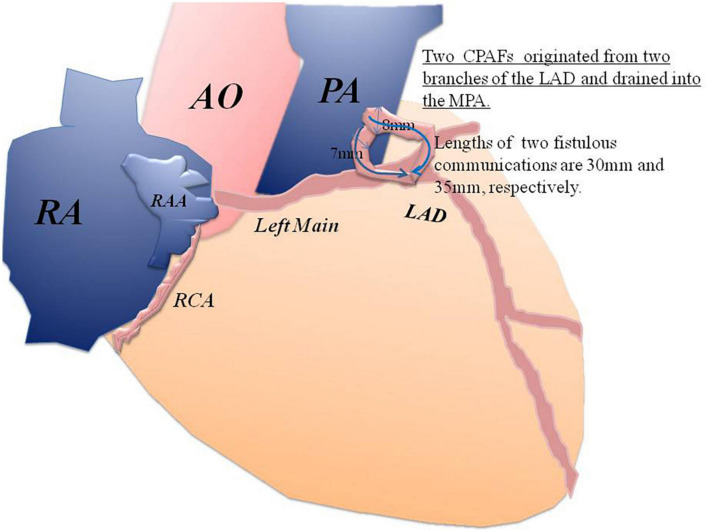
A sketch of the two CPAFs that originated from two branches of the LAD and drained into the MPA.

In a small incision, taking surgical photos was inconvenient and increased the risk of contamination of the surgical field. In this situation, we prioritized patient safety and did not take representative intraoperative photographs. However, we do believe that the rare variant should be reported. To give readers a better understanding, we have provided a sketch (Figure 4).

## Conclusion

We report a rare and complex case with two coronary-pulmonary artery fistulas from two branches of the LAD to the MPA, ASD, and a bicuspid pulmonary valve. Furthermore, it was successfully diagnosed and repaired under OPBHS. The patient was asymptomatic and the small coronary-pulmonary artery fistula might be clinically missed. Therefore, the use of OPBHS to directly detect, evaluate and treat CPAFs is relevant. OPBHS as a myocardial protection strategy is feasible for patients with low surgical risk. The precise role of OPBHS in myocardial protection needs further study.

## Data availability statement

The original contributions presented in this study are included in the article/supplementary material, further inquiries can be directed to the corresponding author.

## Ethics statement

The studies involving human participants were reviewed and approved by the Medical ethics committee of Yichang Central People’s Hospital. The ethics committee waived the requirement of written informed consent for participation.

## Author contributions

All authors listed have made a substantial, direct and intellectual contribution to the work, and approved it for publication.

## Abbreviations

CPAF: Coronary artery to pulmonary artery fistula
LAD: left anterior descending coronary
PA: pulmonary artery
ASD: atrial septal defect
OPBHS: on-pump beating heart surgery
ECG: electrocardiogram
CRBBB: complete right bundle branch block
TR: tricuspid regurgitation
TTE: Transthoracic echocardiography
TEE: transesophageal echocardiography
CPB: cardiopulmonary bypass
I/R injury: ischemia and reperfusion injury
NYHA: New York Heart Association
CABG: coronary artery bypass grafting.
